# Determination of β-Galactooligosaccharides (GOS) in Infant Formula: Collaborative Study, Final Action 2021.01

**DOI:** 10.1093/jaoacint/qsae031

**Published:** 2024-04-13

**Authors:** Denis Cuany, Sean Austin

**Affiliations:** Société des Produits Nestlé S.A., Nestlé Research, Route du Jorat 57, 1000 Lausanne 26, Switzerland; Société des Produits Nestlé S.A., Nestlé Research, Route du Jorat 57, 1000 Lausanne 26, Switzerland

## Abstract

**Background:**

We previously published a method for the determination of β-galactooligosaccharides (GOS) in infant formula and adult nutritionals, which is currently First Action AOAC Method **2021.01**. In this study, reproducibility data were collected to support the promotion of the method to Final Action.

**Methods:**

A collaborative study was organized in which 14 laboratories from eight different countries participated. Initially, laboratories were requested to analyze two practice samples and request guidance from the study director in case of issues. Successful laboratories proceeded to analyze seven samples (six infant formula and one adult nutritional) received as blind duplicates.

**Results:**

Thirteen laboratories reported acceptable results for practice sample 1. Practice sample 2 could only be delivered to eight of the laboratories due to restrictions at customs. The 13 laboratories successfully analyzing practice sample 1 were requested to continue with the analysis of the multilaboratory trial (MLT) samples. Laboratory 14 was unable to solve some technical difficulties, so their data could not be used. Out of the seven samples tested, results for six infant formulas met the requirements of the AOAC *Standard Method Performance Requirements* (SMPR^®^) 2014.003, with repeatability (RSD_r_) ranging from 1.4 to 4.7% and reproducibility (RSD_R_) ranging from 8.1 to 11.6%. The adult nutritional sample returned results outside the range of the SMPR, having an RSD_r_ of 9.9%, higher than the SMPR target of ≤6%, and an RSD_R_ of 12.1%, just above the SMPR target of ≤12%.

**Conclusion:**

The method described is suitable for the determination of GOS in infant formula.

**Highlight:**

A method is described which is suitable for the determination of GOS in infant formula.

β-Galactooligosaccharides (GOS), also known as trans-galactooligosaccharides, are typically produced by the transferase activity of β-galactosidase on lactose (1, 2). This produces a complex mixture of non-digestible oligosaccharides having degrees of polymerization typically between 2 and 7. Determination of GOS in food products can be carried out by the method of de Slegte (3) in which the GOS are hydrolyzed to their component monosaccharides and the released galactose is used to calculate the GOS content. This method is described in detail in AOAC Method **2001.02** (4). Unfortunately, the method is not suitable for the determination of GOS in products with a high background of lactose or galactose (such as dairy products and infant formula). To overcome that limitation, we developed a method based on profiling the oligosaccharides after labelling with 2-aminobenzamide assuming equimolar response factors for all the oligosaccharides (5). However, the method lacked specificity for GOS, therefore further adaptation was made to address that limitation (6) and the method was adopted as First Action AOAC Method **2021.01** (7). To promote the method to a Final Action method, a multilaboratory study is required to assess the between-laboratory method precision. The multilaboratory study is herein described, as well as an adaptation of the method to remove the toxic reagent sodium cyanoborohydride and replace it with the less toxic 2-methylpyridine borane complex (8, 9).

## Collaborative Study Description

A request to participate in a collaborative study was sent to 23 laboratories. Sixteen laboratories in 10 different countries responded positively to the request. After receiving the samples two laboratories were unable to participate due to lack of resources. Finally, 14 laboratories from eight different countries participated and returned results. The study protocol was designed in two parts: *(1)* method setup and qualification of participants and *(2)* multilaboratory trial (MLT) participation.

The MLT sample kit was prepared by the study director. The kit comprised seven commercial products from different suppliers (Table 1). For each powdered infant formula, several containers with the same batch number were combined and homogenized in a large bag to produce a batch of at least 5.6 kg of powder. Pouches of 100 g were prepared and then randomly divided into two groups to obtain blind duplicates. For the three ready-to-feed samples, 48 bottles of 200 mL with the same lot number were collected for each type of sample. All commercial labels were removed. The bottles were separated into two groups at random to obtain blind duplicates. A homogeneity study was performed on six containers of each product (three of each blind duplicate). The First Action AOAC Method **2021.01** protocol was applied to determine the GOS content of the samples. The homogeneity of the sample was assessed based on the GOS content. Statistical analysis was performed using the Cochran C test according to ISO 5725:2020 (10). The calculated Cochran test values were evaluated against ≤1.0% significance. On this basis, all seven samples were found to be homogeneous. The kits of samples were shipped at ambient temperature. The participants were asked to keep the samples at room temperature before undertaking analysis and, once opened, to keep them in tightly closed containers for the duration of the study. The liquid products had to be kept at 4°C once opened. All powdered samples required analysis on a reconstituted basis, using 25 g material and 200 g water. The liquid samples were analyzed “as is”.

**Table 1.** Sample list and coding

Sample details	Information sent to labs	
	Code	Reconstitution rate
RTF formula with GOS/FOS^a^ (PBK-00004)	21–001	As is
IF powder with GOS/HMO^b^ (PBK-00001)	21–002	25 g + 200 g water
RTF adult nutritional (PBK-00007)	21–003	As is
IF^c^ powder (PBK-00002)	21–004	25 g + 200 g water
Organic RTF^d^ formula with probiotics (PBK-00005)	21–005	As is
IF powder with GOS/HMO (PBK-00001)	21–006	25 g + 200 g water
RTF adult nutritional (PBK-00007)	21–007	As is
IF powder with partially hydrolyzed protein (PBK-00006)	21–008	25 g + 200 g water
RTF formula with GOS/FOS (PBK-00004)	21–009	As is
IF powder with GOS/FOS (PBK-00003)	21–010	25 g + 200 g water
Organic RTF formula with probiotics (PBK-00005)	21–011	As is
IF powder (PBK-00002)	21–012	25 g + 200 g water
IF powder with GOS/FOS (PBK-00003)	21–013	25 g + 200 g water
IF powder with partially hydrolyzed protein (PBK-00006)	21–014	25 g + 200 g water

a GOS/FOS = β-Galactooligosaccharides/Fructooligosaccharides.

b HMO = Human milk oligosaccharides.

c IF = Infant formula.

d RTF = Ready to feed.

Two practice samples, one from NIST, United States (PS-1) and one from Global Proficiency, New Zealand (PS-2) were sent before (or in parallel to) the MLT kit. Eight participants received both practice samples. Six participants received only the PS-1 sample, due to issues getting the PS-2 sample through customs. The participating laboratories were asked to analyze the practice samples in duplicate and to report any deviation from the method described in the protocol. The final results were reported in g/100 g of reconstituted product. Participants were also asked to provide sample weights, dilutions, and peak areas of standards and sample extracts to the study director by filling out the reporting template provided with the protocol. Information on the analytical column and instrument type used as well as the brand of maltotriose and its purity were also requested. After review by the study director, the results found to be within a range of expected levels qualified the laboratories for the second part of the study.

All qualified participants were asked to analyze the 14 coded MLT samples (one single determination per sample). Results were communicated to the study director by filling out a reporting template provided with the protocol. The information to be provided in this template was similar to the one used for the practice samples.

All data received were subjected to statistical analysis as described in “Appendix D*: Guidelines for Collaborative Study Procedures To Validate Characteristics of a Method of Analysis*” in the AOAC *Official Methods of Analysis*^SM^ (11) and by using the Excel sheet “AOAC INTERNATIONAL Interlaboratory Study Workbook, Blind Replicates, Version 2.1” (12). Outliers were detected using Cochran and single Grubbs tests in the workbook. The average concentrations, RSD of repeatability (RSD_r_), RSD of reproducibility (RSD_R_) as well as Horwitz ratio values were estimated from the blind duplicates.

***AOAC Official Method* ^SM^ 2021.01**

**β-Galactooligosaccharides (GOS) in Infant Formula**

**First Action 2021**

**Final Action 2023**

[Applicable to the measurement of GOS in infant formula (powders, ready-to-feed liquids, and liquid concentrates) from 0.2 g/100 g to 3.0 g/100 g on a ready-to-feed basis.]

*Caution*: The method employs corrosive, toxic (acute and irritant), and flammable chemicals such as acetic acid, sodium hydroxide, formic acid, ammonia solution, and acetonitrile. Amyloglucosidase is a health hazard and ammonia solution is also dangerous for the environment. Wear personal protective equipment such as gloves and safety glasses. Perform all manipulations under a fume hood. Refer to the materials safety data sheets, take appropriate additional safety precautions for handling, and ensure waste disposal is in accordance with local requirements.

### A. Principle

Powdered or concentrated test samples are reconstituted in water and heated at 70°C for analysis on a ready-to-feed (RTF) basis. Duplicate aliquots of the RTF sample are taken, and both are treated with amyloglucosidase to hydrolyze any maltooligosaccharides present (Assay 1). One aliquot is also treated with β-galactosidase (Assay 2) to hydrolyze all the GOS present. An internal standard (laminaritriose) is added to both aliquots and the oligosaccharides are fluorescently labeled with 2-aminobenzamide (2AB). Labeled extracts are diluted with acetonitrile prior to injection on an ultra-high-performance liquid chromatography (UHPLC) system equipped with a fluorescence detector (FLD) and a hydrophilic interaction LC (HILIC) analytical column. The analytes are separated using a gradient of aqueous ammonium formate in acetonitrile and detected with an FLD. An external maltotriose calibration curve is prepared in the same way as the samples but without enzymatic treatment. Since it is the 2AB label that is detected, each oligosaccharide has an equivalent molar response. The maltotriose calibration curve can thus be used to determine the molar concentrations of the oligosaccharides in the two assays. It is then necessary to know the molecular mass of each signal in the chromatogram to convert the molar concentrations to mass concentrations. This can be done by coupling a mass spectrometer (but once a GOS ingredient profile has been characterized by HILIC–FLD–MS, future samples can be analyzed without the MS). They may also be estimated by comparing the relative retention time (RRT) of the oligosaccharide against that of a dextran ladder, similar to the approach used for glycan analysis (13). The GOS content is obtained by subtracting the oligosaccharide (OS) content obtained in Assay 2 from the OS content obtained in Assay 1.

### B. Apparatus and Materials

- *Analytical balance*.—Weighing to ±0.1 mg (Mettler-Toledo, Greifensee, Switzerland).
- *Weighing boats.*
- *Volumetric flasks.—*10 to 1000 mL.
- *Glass tubes.*—10 or 20 mL.
- *pH meter.—*Reading 0.1 pH (Metrohm, Herisau, Switzerland).
- *Microcentrifuge tubes.—*1.5 mL, safe lock or screw cap (Eppendorf, Hamburg, Germany).
- *Microcentrifuge tubes.*—2 mL, safe lock or screw cap (Eppendorf).
- *Floating rack.*—For microtubes (Nalgene, Thermo Fisher Scientific, Waltham, MA, USA).
- *Water bath.—*At 70 ± 1°C (Thermo Fisher Scientific).
- *Water bath.*—At 65 ± 1°C (Thermo Fisher Scientific).
- *Water bath.—*At 60 ± 1°C (Thermo Fisher Scientific).
- *Centrifuge.—*For 1.5 and 2 mL microtubes able to operate at 10 000 × *g* (Eppendorf).
- *Micropipettes with tips.—*0.02 to 10 mL (Socorex Isba, Ecublens, Switzerland).
- *Vortex mixer.*—Scientific Industries (Bohemia, NY, USA).
- *Ultrasonic bath.*
- *UHPLC column.*—Acquity UPLC BEH Glycan, 1.7 µm; 2.1 mm × 150 mm (Waters, Milford, MA, USA).
- *Liquid chromatography instrument.*—Equipped with a gradient pump able to deliver a flow of 0.3 to 0.6 mL/min with a back-pressure up to 15 000 psi, an online degasser, an autosampler equipped with a refrigerated sample compartment, a temperature-controlled column compartment able to maintain a stable temperature of 25.0 ± 1.0°C, and an FLD (Thermo Fisher Scientific).
- *Mass spectrometer (optional).*—Equipped with an electrospray ionization source able to handle a flow rate of up to 0.4 mL/min and operating at a mass-to-charge ratio between 400 and 1500.

### C. Chemicals and Reagents

- *Deionized water.—*18 MΩ Milli-Q (Merck Millipore, Darmstadt, Germany) purified or equivalent.
- *Maltotriose (with accurately known purity).—*For example, Ultrapure (Carbosynth, Newbury, United Kingdom). In case of issues, check the moisture content and purity following the procedure described in *Annex A*.
- *Laminaritriose*.—>90% (Megazyme, Bray, Ireland).
- *Glacial acetic acid anhydrous.—*GR for analysis (Merck Millipore).
- *Sodium hydroxide pellets.—*Merck Millipore.
- *Acetonitrile.—*Gradient grade for LC (Merck Millipore).
- *Dimethylsulfoxide (DMSO).—*Puriss p.a. (Sigma-Aldrich, St. Louis, MO, USA).
- *2-Aminobenzamide (2AB).—*>98% (TCI Europe, Zwijndrecht, Belgium).
- *2-Methylpyridine borane complex.—*95% (Sigma-Aldrich).
- *Amyloglucosidase (Aspergillus niger).—*9 U/mg (Roche Diagnostics, Rotkreuz, Switzerland: 11 202 367 001).
- *β-Galactosidase (Aspergillus niger).—*4000 U/mL (Megazyme E-BGLAN).
- *Formic acid.—*GR for analysis (Merck Millipore).
- *Ammonium hydroxide solution 25–30%.—*GR for analysis (Merck Millipore).
- *Dextran.—*With average MW 1000 Da (Fluka, Darmstadt, Germany).
- *Isomaltose.—*Carbosynth, Compton, United Kingdom.

### D. Preparation of Reagents

- *Maltotriose (malto-3) stock solution (about 10 µmol/mL).—*Weigh 100 mg maltotriose into a weighing boat and record the mass to 0.1 mg. Transfer quantitatively into a 20 mL volumetric flask with water and dilute to volume with the same solvent.
- *Laminaritriose internal standard working solution (about 2 µmol/mL).—*Weigh the whole content of a 50 mg laminaritriose vial into a weighing boat and record the mass to 0.1 mg. Transfer quantitatively into a 50 mL volumetric flask and complete to the mark with water.
- *Sodium hydroxide (1 M)—*Dissolve 10 ± 0.2 g sodium hydroxide pellets in 200 mL water in a 250 mL volumetric flask. After cooling to room temperature, make up to the mark with deionized water and mix well.
- *Sodium acetate buffer (0.2 M, pH 4.5).—*Into a large beaker (>500 mL) containing 400 mL deionized water, pipette 5.8 mL glacial acetic acid. Adjust to pH 4.5 with sodium hydroxide solution 1 M. Transfer the solution to a 500 mL volumetric flask and make up to the mark with water.
- *Water–acetonitrile solution (25 + 75).—*Add 50 ± 1 mL water to 150 ± 1 mL acetonitrile in a glass bottle and mix.
- *2AB labeling reagent.—2AB (0.35 mol/L)–2-methylpyridine borane complex (1.0 mol/L) in DMSO–acetic acid (70 + 30) solution.*—Pipette the volume of DMSO and glacial acetic acid in a 20 mL glass tube according to the number of tests to perform (*see*  Table **2021.01A** for example quantities). Mix the solution using a vortex mixer. Weigh the amount of 2AB and 2-methylpyridine borane complex (*see*  Table **2021.01A**) in another glass tube of 20 mL, then add the corresponding volume of 30% acetic acid in DMSO. Mix using a vortex mixer and use an ultrasonic bath for complete dissolution (about 10 min).
- *Amyloglucosidase solution (60 U/mL in 0.2 M sodium acetate buffer pH 4.5).—*Weigh an amount of amyloglucosidase corresponding to 600 ± 20 U and dissolve with 10.0 mL sodium acetate buffer 0.2 M, pH 4.5. This solution is prepared on the day of use and kept at 4°C until use.
*Note:* For the development and validation of this method, amyloglucosidase (Cat. No. 11202367001) from Roche Diagnostics, was used. Enzyme activities may vary slightly from one batch to the other (units/mg are mentioned on the label). Adapt the weight of enzyme in order to reach a concentration of 60 ± 6 U/mL. Another amyloglucosidase, (Cat. No. 11202367001), also available from Roche Diagnostics, has also been tested and found to be suitable. This enzyme is already in suspension (140 U/mL) and can be diluted with 0.2 M sodium acetate buffer pH 4.5 in order to produce a working concentration (60 U/mL). When enzymes from another source are used, it is imperative to ensure the enzymes employed will completely hydrolyze any maltodextrins in the product without hydrolyzing any analytes, as well as not adding any interference in the chromatogram. This can be checked by performing an analysis with maltodextrin as a sample, a GOS ingredient as a sample, and running a blank with the amyloglucosidase only.
- *β-Galactosidase solution (4000 U/mL).—*Use the solution as is.
*Note*: For the development and validation of this method, the β-galactosidase E-BGLAN, available from Megazyme, was used. When enzyme from another source is used, it is imperative to ensure the enzyme employed will completely hydrolyze the GOS without hydrolyzing any other oligosaccharides that may be present in the sample.
- *Dextran solution.—*Weigh about 20 mg isomaltose and about 50 mg dextran 1000 into a weighing boat. Transfer into a 50 mL volumetric flask with water and dilute to volume with the same solvent.

**Table 2021.01A.** Examples of quantities for 2AB reagent preparation

	30% Acetic acid in DMSO		0.35 M 2AB + 1 M 2-methylpyridine borane complex in 30% acetic acid in DMSO		
Max. number of tests	DMSO, mL	100% Acetic acid, mL	30% Acetic acid in DMSO, mL	2AB, mg	2-Picoline borane, mg
50	4.7	2.0	6.00	286 ± 5	642 ± 10
100	11.6	5.0	12.5	596 ± 10	1337 ± 15
250	23.3	10.0	30.0	1430 ± 15	3209 ± 15

### E. Mobile Phase Preparation

- *Eluent A.—*Acetonitrile.
- *Eluent B.—*Ammonium formate (100 mM, pH 4.4). Add 4.6 ± 0.1 g (3.78 mL) formic acid (100%) in a beaker containing 800 mL water. Adjust the pH to 4.40 ± 0.05 with ammonium hydroxide solution (25–30%). Transfer quantitatively to a 1000 mL volumetric flask and dilute to volume with water.

### F. Preparation of Standards

Prepare a 6-level calibration curve by diluting the maltotriose stock solution as described in Table **2021.01B**, using volumetric flasks made up to the final volume with water.

**Table 2021.01B.** Dilution scheme for the preparation of the standard curve

Standard	Volume of maltotriose stock solution, µL	Final volume, mL	Maltotriose concentration, µg/mL^a^
Level 1	200	100	20
Level 2	400	20	200
Level 3	800	20	400
Level 4	1200	20	600
Level 5	1600	20	800
Level 6	2000	20	1000

a This is the nominal concentration, calculate the actual concentration based on the actual concentration of stock solution prepared in the laboratory and adjusted for the purity and moisture content of the standard being used.

### G. Sample Preparation

- *For analysis of products on a RTF basis reconstitute powder or liquid concentrates according to manufacturer instructions.—*(e.g., weigh 25 g of an infant formula powder into a bottle and add water to a final total weight of 225 g). Place the mixture in a water bath at 70°C for 25 min under constant stirring. Cool the solution to room temperature.
- *For reconstituted products (as prepared above), or products which are sold as RTF*.—Weigh an amount of test sample (*m*) containing a maximum of 50 mg GOS but not more than 5 g of test sample, into a 25 mL (*V*) volumetric flask and make up to the mark with water.
- *For analysis of homogeneous powder products without prior reconstitution*.—Weigh an amount of test sample (*m*) containing a maximum of 100 mg GOS but not more than 1.1 g of powder, into a 50 mL (*V*) volumetric flask. Add water (30 mL) and heat at 70°C with constant agitation for 25 min. Cool to room temperature and complete to the mark with water.
- *Standard calibration curve.—*With each series of analyses, prepare a maltotriose calibration curve (6-level, Table **2021.01B**). For each of the calibration standards, transfer 500 µL into a microtube (1.5 mL). Add 250 µL water. Mix on a vortex mixer and place in a water bath at 60°C for 2 h. At the end of the incubation time, mix on a vortex mixer and place at 4°C for 5–10 min and then continue with the standards from step **[G(h)]**.
- *Dextran ladder.—*With each series of analyses, prepare a dextran ladder. Into a microtube (1.5 mL), transfer 500 µL dextran solution **[D(i)]**. Add 250 µL water, mix on a vortex mixer and then continue with the dextran ladder from step **[G(h)]**.
- *Hydrolysis of maltodextrins (Assay 1 and Assay 2) and GOS (Assay 2).—*Into two microtubes (1.5 mL) marked A1 (Assay 1) and A2 (Assay 2), transfer 500 µL test solution. Add 200 µL amyloglucosidase solution (60 U/mL in 0.2 M sodium acetate buffer pH 4.5) in both tubes. Add 50 µL water in the tube marked A1 and 50 µ β-galactosidase solution in the tube marked A2. Mix on a vortex mixer and place in a water bath at 60°C for 2 h ± 5 min. At the end of the incubation time, put all tubes with β-galactosidase (Assay 2) in a boiling water bath for 5–6 min to stop the reaction. Then mix on a vortex mixer and place at 4°C for 5–10 min.
- *Reagent blank.—*With each series of analyses, prepare a reagent blank by performing the whole procedure on water instead of the test solution (Assay 1 and Assay 2).
- *Internal standard addition.—*Centrifuge all tubes (standard curve, Assay 1, Assay 2, Blank 1, Blank 2, and dextran ladder) for 10–20 s at 10000 × *g* to remove drops from the lid. Add 100 µL laminaritriose (2.0 µmol/mL) to all tubes and mix well on a vortex mixer.
- *Derivatization.—*Transfer 20 µL of treated solutions containing internal standard into 2 mL microtubes (safe lock or screw cap), add 100 µL water and 100 µL 2AB labeling reagent to each tube. Mix on a vortex mixer and place the tubes in a water bath at 65°C ± 1°C for 1 h ± 5 min. After 1 h mix the tubes on a vortex mixer then place at 4°C for 5–10 min.
- *Dilution.—*Once cooled, centrifuge for 10–20 s at 10000 × *g* to remove drops from the lid. Carefully open the microtubes under a fume hood and dilute by addition of 1 mL acetonitrile–water (75 + 25) solution. Mix well on a vortex mixer then centrifuge for 5 min at 10000 × *g* before transferring 1 mL supernatant to an injection vial.

### H. Chromatographic Conditions

The UHPLC system is equipped with an Acquity UPLC BEH Glycan column (2.1 mm × 150 mm, 1.7 µm). The column is held at 25 ± 1°C and the injection volume is 2 µL. The analytes are separated using the gradient described in Table **2021.01C** and are detected by means of an FLD set at the following wavelengths: excitation λ = 330 nm and emission λ = 420 nm.

**Table 2021.01C.** UHPLC gradient for separation of oligosaccharides

Time, min	Flow rate, mL/min	Acetonitrile, % A	Ammonium formate, 100 mM, pH 4.4, % B
0.0	0.6	88.0	12.0
7.0	0.6	88.0	12.0
17.0	0.6	85.0	15.0
21.0	0.6	85.0	15.0
36.0	0.6	72.6	27.4
44.0	0.6	54.0	46.0
44.1	0.3	54.0	46.0
45.5	0.3	30.0	70.0
49.5	0.3	30.0	70.0
52.0	0.3	88.0	12.0
54.0	0.6	88.0	12.0
60.0	0.6	88.0	12.0

### I. System Suitability Test

Before starting an analysis allow the chromatographic system to equilibrate and the fluorescence detector to warm up (if necessary) under the initial conditions, for at least 15 min. Let the derivatized standard and sample solutions equilibrate to the autosampler temperature for 10–15 min before making any injections. Ensure the system pressure and baseline are stable and there are no leaks. Before starting a series of analyses, make at least one injection of the dextran ladder, and check the relative retention times (RRT) of the oligosaccharides compared to laminaritriose. The RRTs should be in the following range: isomaltose 0.56–0.60, isomaltotriose 1.50–1.57, isomaltotetraose 1.92–2.12, isomaltopentaose 2.16–2.40, and isomaltohexaose 2.29–2.58. If the RRTs are outside this range it may be indicative that the analytical column needs replacement or that there is a problem with the mobile phase (either with the preparation of the ammonium formate, or with the eluent mixing in the LC system).

### J. Calibration and Calculations

It is recommended to use bracketed calibration, injecting three standards followed by a maximum of 10 samples then three standards, etc. For example, inject standards at levels 1, 3, and 5 then 10 samples, then standards at levels 2, 4, and 6, then 10 samples, then standards 1, 3, and 5, etc. Use the instrument software to plot a 6-point standard curve of “instrument response for maltotriose/instrument response for laminaritriose” against the “concentration of maltotriose” in the standard (in µmol/mL). Fit a linear model to the data including the origin as a point (but not forced through the origin).

When integrating the peaks in the chromatogram it is important to make an estimate of the S/N of the smaller peaks. Only include peaks with an S/N of 10 or greater in the calculations. Smaller peaks cannot be quantified accurately and introduce inaccuracies in the measurement if included. Do not integrate any peaks smaller than disaccharides. Do not integrate the lactose peak or the maltose peak (if present). In Assay 2, a peak may appear in the region which was covered by the lactose signal in Assay 1: do not integrate this peak. Beware that some human milk oligosaccharides (HMOs) that contain a terminal galactose at the non-reducing end (e.g., lacto-N-tetraose (LNT), lacto-N-neotetraose (LNnT), lacto-N-hexaose (LNH), etc.) can be partially hydrolyzed by β-galactosidase treatment. If present, they will appear to have a smaller molecular mass in Assay 2 compared to Assay 1. Signals from such HMOs should be identified correctly and assigned the same molecular mass in both assays. In case of doubt, run the HMO through the method and the peaks concerned can be identified.

Use the standard curve to calculate the molar concentration ($C_{mA}$_,_ in µmol/mL) of each oligosaccharide in the chromatogram without β-galactosidase treatment (Assay 1) and calculate the total oligosaccharides in that sample using Equation 1. Then use the standard curve to calculate the molar concentration ($C_{mB}$, in µmol/mL) of each oligosaccharide in the chromatogram after β-galactosidase treatment (Assay 2) and calculate the total oligosaccharides in that sample according to Equation 2. Then calculate the GOS content of the sample using Equation 3. If calculation of the dietary fiber content of the GOS is desired, exclude the disaccharides from the calculations in Equations 1 and 2.

(1) $$C_{\mathrm{TOS}}=\sum (C_{mA}\;\times \;MW)\;\times \;\frac{V}{m}\;\times \;0.0001$$

 

(2) $$C_{B}=\sum (C_{mB}\;\times \;MW)\;\times \;\frac{V}{m}\;\times \;0.0001$$

 

(3) $$C_{\mathrm{GOS}}=C_{\mathrm{TOS}}-\;C_{B}$$

where $C_{\mathrm{TOS}}$ = total concentration of oligosaccharides in the untreated sample (in g/100 g); $C_{B}$ = total concentration of oligosaccharides in the enzyme-treated sample (in g/100 g); $C_{\mathrm{GOS}}\;$= total concentration of GOS in the sample (in g/100 g); $C_{mA}$ = molar concentration of each individual oligosaccharide in the untreated sample (in µmol/mL); $C_{mB}$ = molar concentration of each individual oligosaccharide in the sample (in µmol/mL) that was enzyme treated; MW = molecular mass of each individual oligosaccharide in the sample, either estimated from the glucose unit (GU) value (see *Annex B*) or measured by mass spectrometry (*Annex C*); V = volume to which the original sample weight was diluted (in mL); m = weight of sample diluted to volume (V; in g); and 0.0001 = factor to convert result from µg/g to g/100 g).

### K. Example Chromatograms

An example chromatogram of the calibration standard is shown in Figure **2021.01A**. Example chromatograms from GOS-containing samples are shown in Figures **2021.01B** and **2021.01C**. Note the change in retention time of LNnT after β-galactosidase treatment (Figure **2021.01C**).

**Figure 2021.01A.**
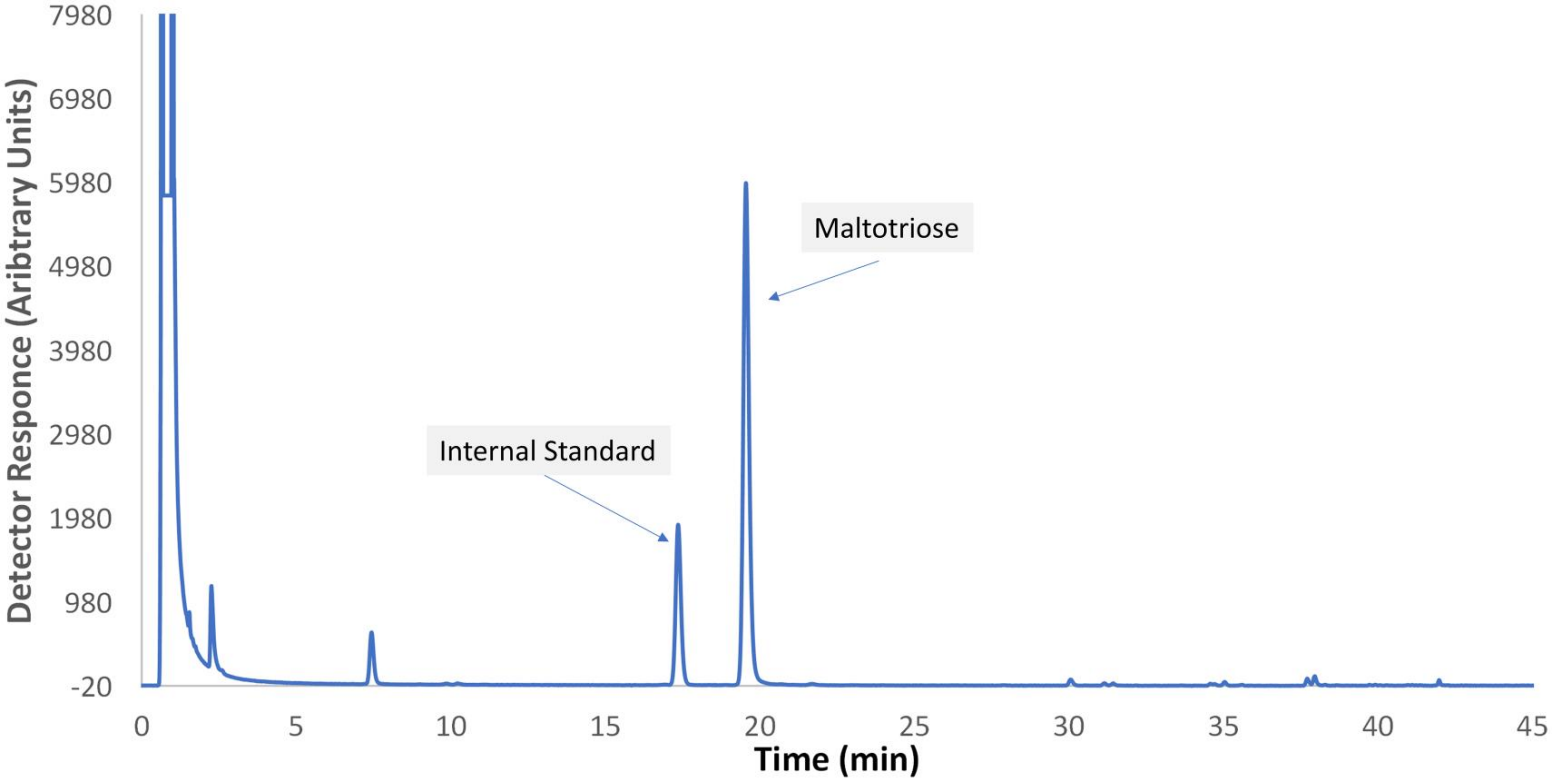
Example chromatogram of standard solution level 6.

**Figure 2021.01B.**
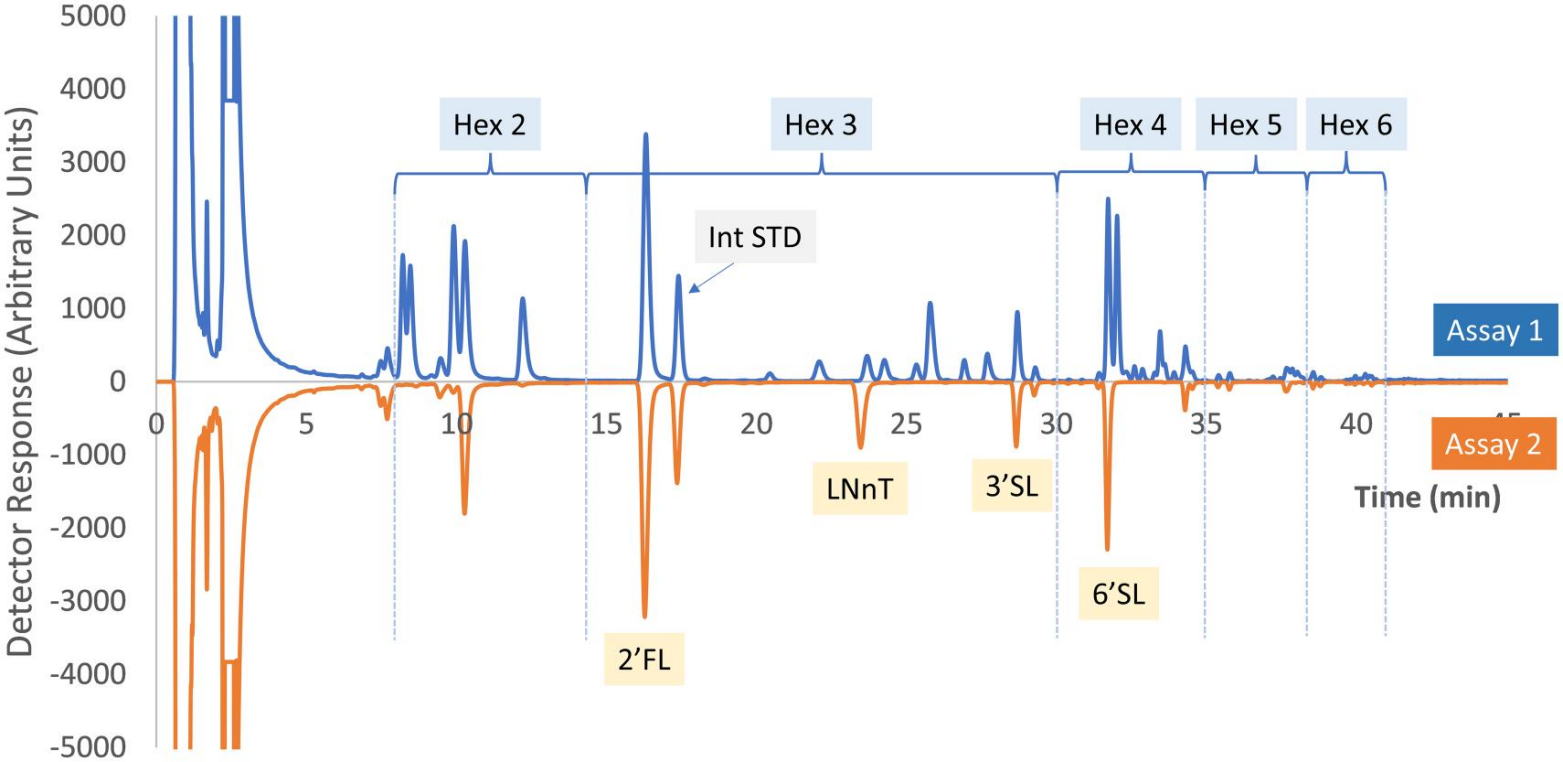
Example chromatograms of infant formula powder with GOS and HMOs (2'-fucosyllactose (2-FL), LNnT, 3'-sialyllactose (3’SL), 6’SL). Hex 2 = disaccharides, Hex 3 = trisaccharides, Hex 4 = tetrasaccharides, Hex 5 = pentatsaccharides, Hex 6 = hexasacchaides.

**Figure 2021.01C.**
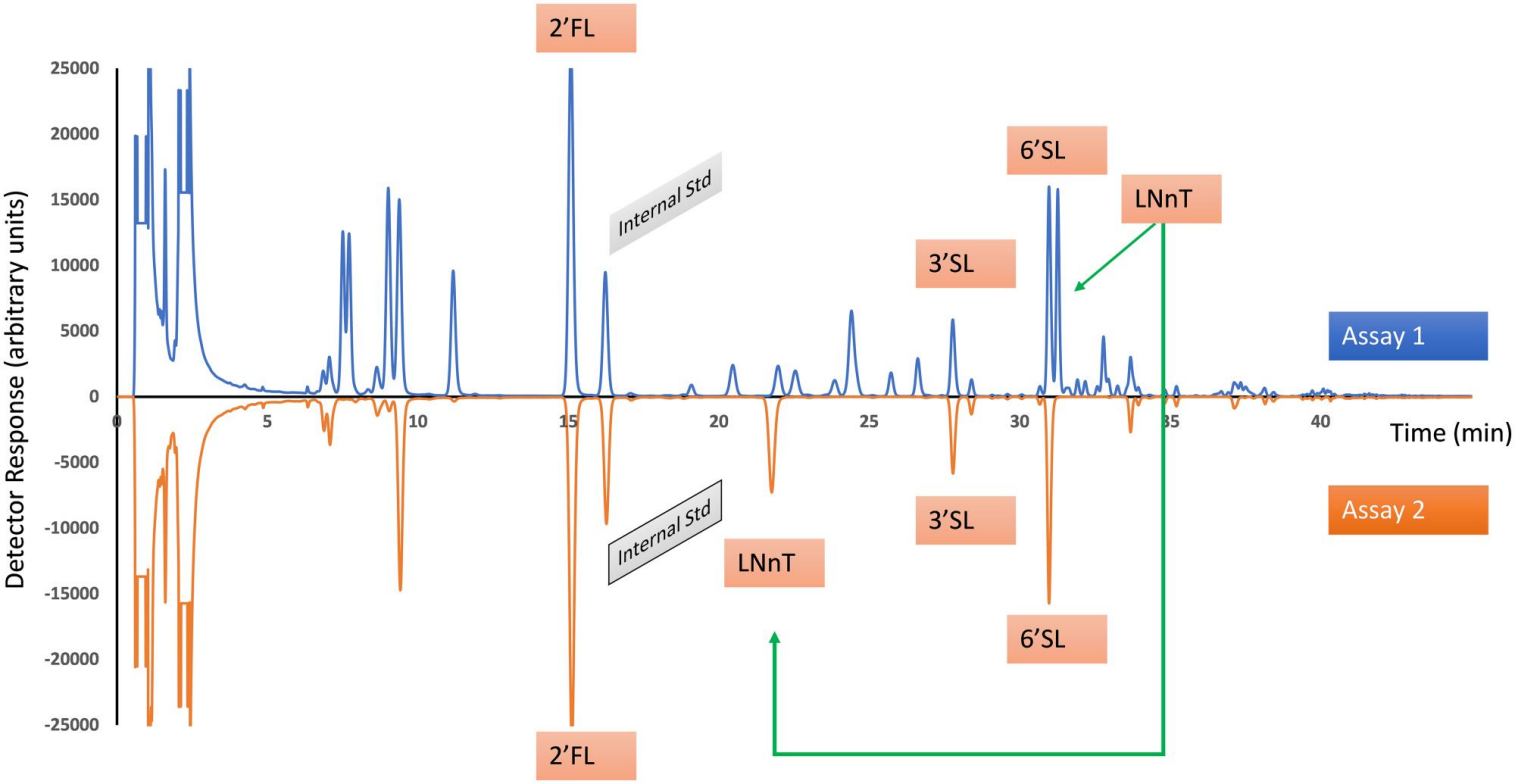
Example chromatograms showing the change in retention time of LNnT due to partial hydrolysis of LNnT.

## AOAC 2021.01. Annex A: Purity Determination of Maltotriose

The purity of the maltotriose is assessed by analyzing the standard prepared at level 6 (expected concentration approximately 1600 nmol/mL). The standard is prepared and injected on the chromatographic system as described in the main method. The chromatogram is then assessed for signals in addition to the ones expected (maltotriose and the internal standard, laminaritriose). The peak areas are measured for all peaks except for laminaritriose and assigned a corresponding molecular mass depending on the identity of the signal [180 for glucose, 342 for saccharides composed of two hexose (Hex) units (Hex 2), 504 for Hex 3 (including maltotriose), 666 for Hex 4, 828 for Hex 5, 990 for Hex 6, and 1152 for Hex 7 (*see*  Figure **2021.01D**)]. The peak area of maltotriose multiplied by its molecular mass (504) is then divided by the sum of all the peak areas multiplied by their corresponding masses to calculate the purity of maltotriose. This is best illustrated by an example. An example data set is shown in Table **2021.01D**. Taking this data, the purity of the maltotriose would be calculated as shown in Equation 4.

(4) $$P_{M3}(\%)=\frac{A_{M3}\;\times \;{MW}_{M3}}{\sum_{i=1}^{n}A_{Mi}\;\times \;{MW}_{Mi}}\;\times \;100=\;\frac{40320}{43362}\;\times \;100=93.0\%$$

where, *P_M3_*(%) = purity of maltotriose in %; *A_M3_* = peak area of maltotriose; *MW_M3_* = molecular mass of maltotriose in g/mol; *A_Mi_* = peak area of signal with i hexose units; *MW_Mi_* = molecular mass of oligosaccharide with i hexose units in g/mol.

**Figure 2021.01D.**
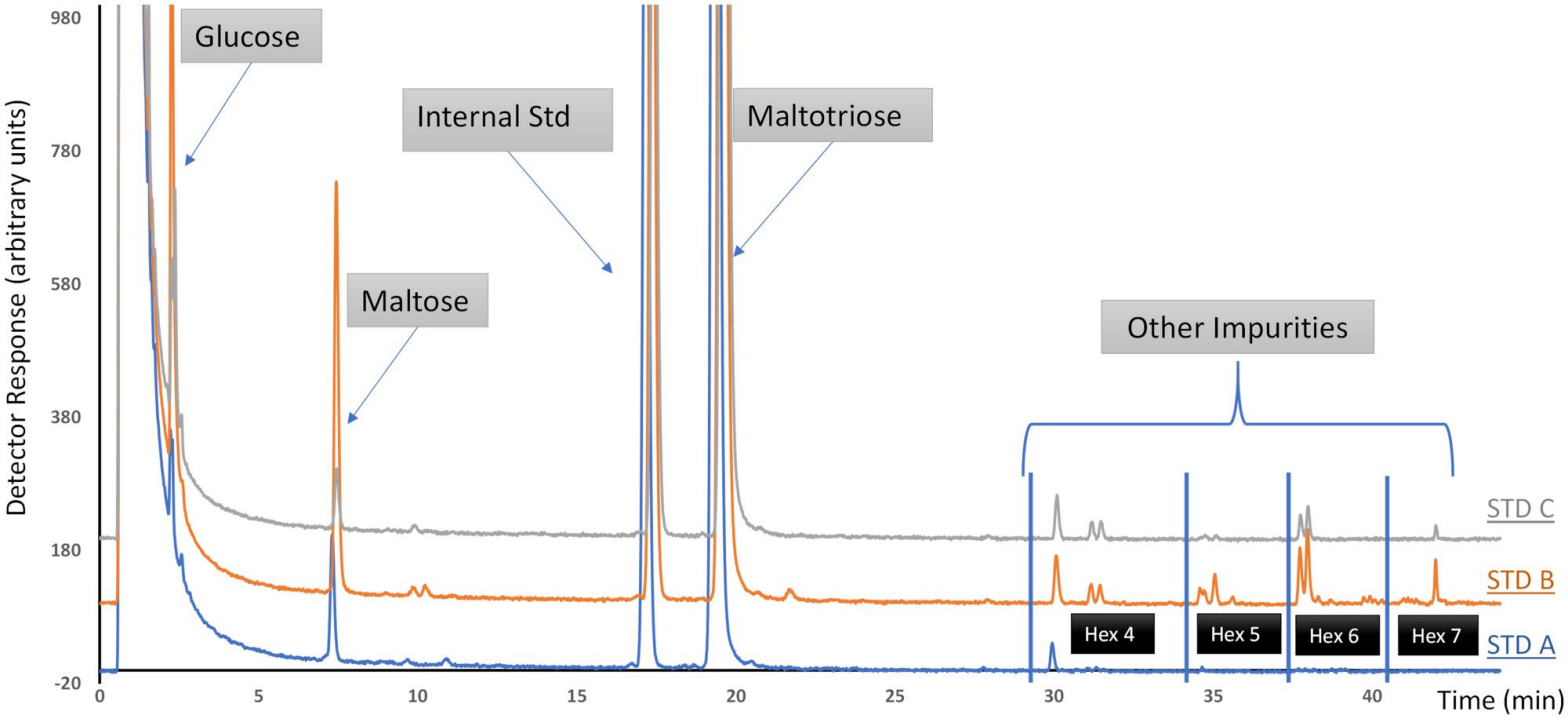
Example chromatograms for maltotriose purity checks.

**Table 2021.01D.** Example data set for illustration of calculation of maltotriose purity

Identity	MW, g/mol	Peak area	Peak area × MW
Glucose	180	2	360
Maltose	342	3	1026
Maltotriose	504	80	40320
Hex 4	666	1	666
Hex 5	990	1	990
Sum	NA^a^	NA	43362

a NA = Not applicable.

If the measured moisture and purity are in good agreement with the manufacturer’s certificate of analysis (CoA) (each ± 2 g/100 g), it is recommended to use the data provided on the manufacturer’s CoA. If the difference is large, it is recommended to use the measured moisture and purity or to use a different batch of maltotriose.

## AOAC 2021.01. Annex B: Determination of Molecular Weight Using Dextran Ladder

It is possible to calibrate the column using the dextran ladder to determine the molecular mass of each signal. The dextran oligosaccharides elute from the smallest to the largest. Isomaltose is the first signal and is composed of two GUs, it is therefore assigned a GU value of 2, the next oligosaccharide of the dextran ladder has a GU of 3, etc., (Figure **2021.01E**).

**Figure 2021.01E.**
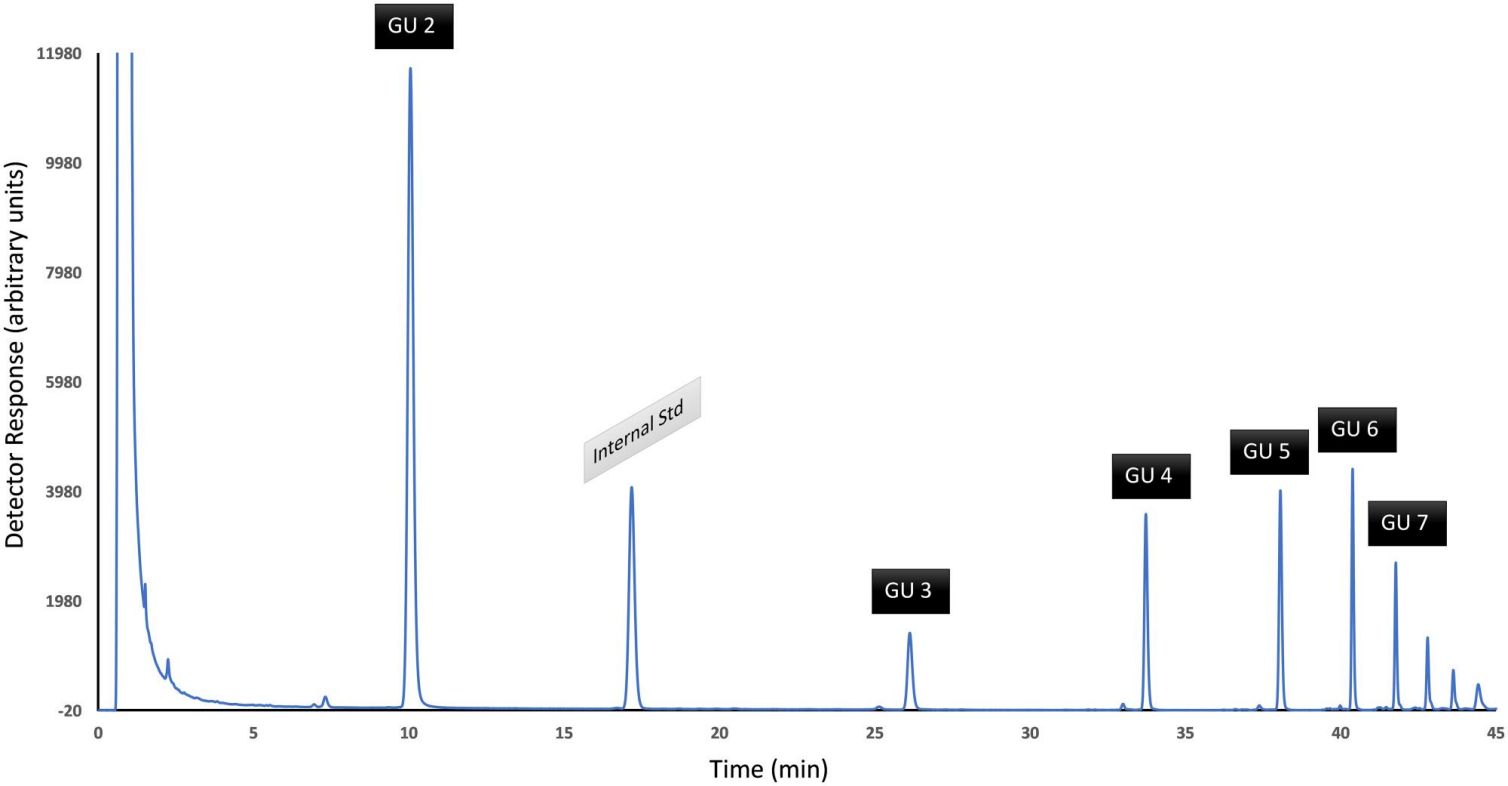
Example chromatogram of dextran ladder.

Determine the RRT of each of the dextran signals compared to the internal standard according to Equation 5. Then make a plot of GU against RRT (only using signals with GU between 2 and 6) and fit a third-order polynomial (y = ax^3^ + bx^2^ + cx + d). For each signal in the sample chromatograms calculate the RRT in the same way as for the dextran ladder, then assign a GU value from the polynomial. The molecular mass of each signal can then be assigned based on the GU value using the information in Table **2021.01E**.

(5) $$\mathrm{RRT}(Dn)=\frac{RT(Dn)}{RT(IS)}$$

where *RRT(Dn)* = relative retention time of the signal n in the dextran ladder; *RT(Dn)* = retention time of signal n in the dextran ladder; and *RT(IS)* = retention time of laminaritriose internal standard.

**Table 2021.01E.** Assignment of peak molecular weight according to its GU value

GU range	GOS type	Molecular mass, g/mol
1.6–2.5	Hex 2	342
2.5–2.6	Internal standard	
2.6–3.4	Hex 3	504
3.4–4.2	Hex 4	666
4.2–5.2	Hex 5	828
5.2–6.2	Hex 6	990
>6.2	Hex 7	1152

As an example, using the data set in Table **2021.01F** one can construct the curve of GU versus RRT (Figure **2021.01F**). From this curve, one can estimate the GUs of peaks in the chromatogram, and eventually the molecular mass using the information from Table **2021.01E**. An Excel workbook has been prepared to automate this process and is available in the Supplemental Information.

**Figure 2021.01F.**
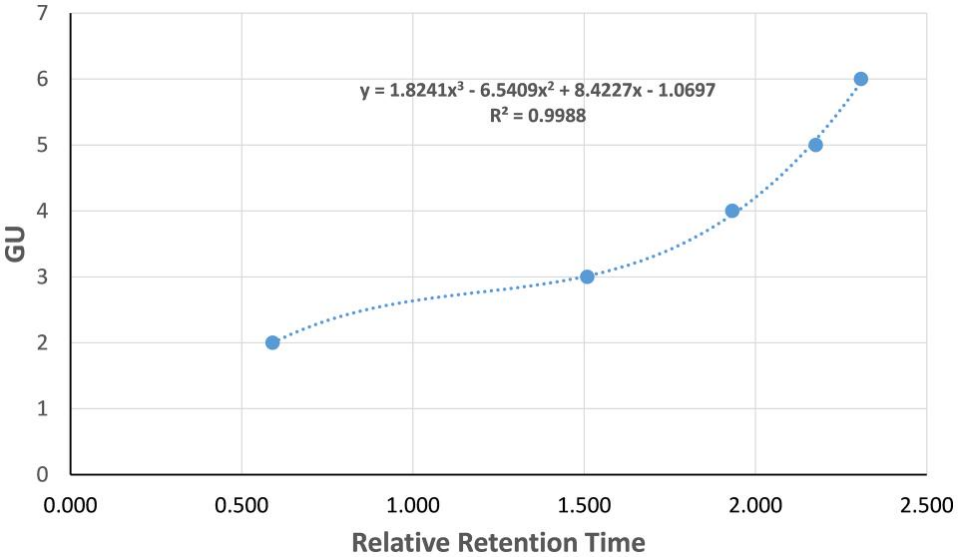
Curve to estimate GU from RRT using example data set in Table 2021.01F.

**Table 2021.01F.** Example data set assigning molecular mass based on GU values

Source	Identity	RT^c^, min	RRT^d^	GU^e^	GOS type^a^	Assigned molecular weight (amu) ^a^
Dextran ladder	Laminaritriose (IS)	17.467	1.000	(IS)^f^	NA^b^	NA
Dextran ladder	Isomaltose	10.317	0.591	2	NA	NA
Dextran ladder	Isomaltotriose	26.359	1.509	3	NA	NA
Dextran ladder	Isomaltotetraose	33.759	1.933	4	NA	NA
Dextran ladder	Isomaltopentose	38.017	2.177	5	NA	NA
Dextran ladder	Isomaltohexose	40.325	2.309	6	NA	NA
GOS	Laminaritriose (IS)	17.501	1.00	(IS)	NA	NA
GOS	Peak A	13.917	0.795	2.409	Hex 2^g^	342
GOS	Peak B	35.034	2.002	4.212	Hex 5^h^	828

a GOS Type and molecular weight assigned from GU value and Table 2021.01E. GOS  =  β-galactooligosaccharides.

b NA = Not applicable.

c RT = Retention time.

d RRT = Relative retention time.

e GU = Glucose units.

f IS = Internal standard.

g Hex 2 = Disaccharide composed of two hexoses.

h Hex 5 = Pentasaccharide composed of 5 hexoses.

## AOAC 2021.01. Annex C: Determination of MW Using LC–FLD–MS

It is possible to determine the molecular mass of each signal in the chromatogram using a mass spectrometer as follows.

*Sample preparation.—*The same vial prepared for the quantitative determination of GOS can also be used for the analysis of MW assignment). Alternatively, one may prepare a sample using only the GOS ingredient. If the mass spectrometer has insufficient sensitivity, it is possible to prepare a sample having 10 times greater GOS concentration for the purposes of peak identification only (in this case it is recommended to use the GOS ingredient).

*Mass spectrometer setup.—*In addition to the UHPLC–FLD instrument, a mass spectrometer is required. Use the same chromatography setup and conditions as described in the main method, but split the flow eluting from the analytical column in a ratio of about 1:1. One half of the flow is passed through the fluorescence detector, the other half is directed to a mass spectrometer (*Note:* if you connect the mass spectrometer in series after the fluorescence detector there is a high chance that the flow cell will rupture).

The following describes the setup of the API 4000 QTrap mass spectrometer in our laboratory. The mass spectrometer settings in other laboratories should be optimized locally.

*LC parameters.—*Use the same LC conditions as described in the quantitative method.

Injection volume can be increased up to 10 µL if necessary to achieve sufficient sensitivity in the mass spectrometer.

*MS parameters.—*Experiment type: Multiple ion monitoring (Q1 or EMS), monitoring the masses listed in Table **2021.01G**.

Mode: Electrospray Ionisation (ESI) negative

Curatin Gas (CUR): 17.0

Ionspray voltage (IS): −3800 V

Gas 1 (GS1): 60.0

Gas 2 (GS2): 20.0

Interface heater Temperature (IHT): 400.0°C

Declustering Potential (DP): −60 V

Entrance Potential (EP): −10.0 V

**Table 2021.01G.** Masses monitored for the assignment of GOS moleular weight

Q1 mass, Da	Dwell time, ms	Corresponding GOS	GOS molecular weight (amu)
461.3	50.0	Hex 2 (including lactose)	342
623.4	50.0	Hex 3	504
785.4	50.0	Hex 4	666
947.4	50.0	Hex 5	828
1109.5	50.0	Hex 6	990
1271.6	50.0	Hex 7	1152
1433.6	50.0	Hex 8	1314

## Results and Discussion

### New Protocol

After the approval of the First Action method, the team of the study director tested the replacement of sodium cyanoborohydride (toxic) by 2-methylpyridine borane complex. A comparison of the methods was carried out to determine whether the alternative method (using 2-methylpyridine borane complex as the reducing agent) is equivalent to the reference method (using sodium cyanoborohydride as the reducing agent). Five samples (Table 2) were analyzed in duplicate on at least six different days. The in-house statistical package Q-Stat was used for comparison of the methods, it uses a *t*-test with a 95% confidence interval to assess if there is a bias between the means of the two methods. The results (Table 3) demonstrate there are no biases between the methods. The precision characteristics of the method also seem to be comparable when using the new protocol (Table 3).

**Table 2.** Samples used for comparison of protocols using different reducing agents

No.	Product description	Sample type
20	Infant formula powder with GOS (1)	Powder
21	Infant formula powder with GOS (2)	Powder
22	Infant formula powder with GOS/FOS	Powder
23	Ready to feed adult nutritional with GOS	Liquid
25	Infant formula powder with GOS/HMO (laboratory reference)	Powder

**Table 3.** Results of method comparison

No.	Sample description	Type of method	Number of replicates (d × *n*)^c^	Mean GOS content, g/100g	RSD_r_, %	RSD_iR_, %	*t*-Test result
20	Infant formula powder with GOS^a^	Alt	(6 × 2)	0.449	0.99	3.68	No bias
		Ref	(6 × 2)	0.437	3.11	4.94	
21	Infant formula powder with GOS^a^	Alt	(6 × 2)	0.279	1.60	4.54	No bias
		Ref	(6 × 2)	0.277	1.33	7.81	
22	Infant formula powder with GOS/FOS^a^	Alt	(6 × 2)	0.773	1.13	2.67	No bias
		Ref	(6 × 2)	0.769	2.50	4.90	
23	Ready to feed adult nutritional with GOS^a^	Alt	(6 × 2)	0.677	2.15	2.43	No bias
		Ref	(6 × 2)	0.664	8.30	9.80	
25	Infant formula powder with GOS/HMO (lab reference)^b^	Alt	(7 × 2)	6.71	1.30	3.8	No bias
		Ref	(7 × 2)	6.52	1.30	2.7	

a Total GOS results in g/100 g product as consumed (reconstituted).

b Total GOS results in g/100 g product on powder.

c d = number of days, *n* = number of replicates per day.

### Practice Sample

The 14 participating laboratories started the method evaluation with the analysis of one or two practice samples (PS-1 and PS-2). PS-1 (SRM 1849 b, NIST, United States) was received by all 14 participants. PS-2 (12067-QC, Global Proficiency, New Zealand) was only received by eight participants due to problems clearing customs. The practice samples were used for laboratories to set up the method and become familiar with the protocol and the provided calculation sheet. Some participants had difficulties with the integration of the peaks and the processing of the results, but after some discussion, they were able to master the method and produce consistent results for the practice samples. One laboratory (Laboratory 14) reported that they had significant problems with interferences in their chromatogram. Unfortunately, they did not have time to resolve this with the study director. Consequently, their data are not included in the statistical evaluation and their results are marked as “unable to report results”. The method was applied in most laboratories without modification. For PS-1, the mean GOS content measured by the 13 laboratories was 0.261 g/100 g on the reconstituted sample, with an RSD_r_ of 2.9% and an RSD_R_ of 10.2%. Since PS-1 was reconstituted as 10 g in a total of 100 g, the GOS content of the powder can be calculated as 2.61 g/100 g, which compares well with the (non-certified) reference value of 2.575 ± 0.055 g/100 g. This indicates that the method achieves the expected results, with good precision. All practice sample results from participants can be found in Table 4. Statistical evaluation of the results from the determination of GOS in both practice samples can be found in Table 5. The practice data from 13 laboratories were considered acceptable and those laboratories were requested to continue with the analysis of the MLT samples.

**Table 4.** Results of practice sample

	PS-1^a^GOS content, g/100g		PS-2^b^ GOS content, g/100g	
Lab code	A	B	A	B
1	0.237	0.240	0.210	0.226
2	0.222	0.226	—^c^	—^c^
3	0.301	0.294	0.241	0.223
4	0.294	0.292	—^c^	—^c^
5	0.295	0.302	0.228	0.235
6	0.256	0.274	0.189	0.195
7	0.272	0.271	0.190	0.185
8	0.263	0.267	0.190	0.200
9	0.267	0.290	—^c^	—^c^
10	0.233	0.216	0.171	0.163
11	0.235	0.233	—^c^	—^c^
12	0.251	0.248	—^c^	—^c^
13	0.261	0.247	0.187	0.177
14	Unable to report results		—^c^	—^c^

a Results are reported in g/100 g of product as reconstituted (10 g product + 90 g water).

b Results are reported in g/100 g of product as reconstituted (25 g product + 200 g water).

c The PS-2 sample could not be delivered to the laboratory.

**Table 5.** Statistical evaluation of results from determination of GOS in the practice sample

Requirements (SMPR 2014.003)			≤6%		≤12%

Sample name	*n^a^*	Mean, g/100 g	RSD_r_, %		RSD_R_, %
PS-1^b^	13 (0)	0.261	2.9		10.3
PS-2^c^	8 (0)	0.201	3.9		12.2

a Number of laboratories considered in the evaluation (number of laboratories with data removed as outliers).

b Results are reported in g/100 g of product as reconstituted (10 g product + 90 g water).

c Results are reported in g/100 g of product as reconstituted (25 g product + 200 g water).

### MLT Samples

The data submitted by the 13 laboratories on the MLT samples were analyzed and the results of the statistical evaluation are shown in Table 6. The full data set per participant are reported in Tables 7 and 8. GOS concentrations in the MLT samples range from 0.23 to 0.86 g/100 g of reconstituted or RTF formula. This corresponds to the lower levels recommended by SMPR 2014.003. The study director was unable to source commercial samples with GOS contents between 1 and 3 g/100 g of reconstituted sample (for infant formula, the maximum permitted dose of GOS in most countries is 0.8 g/100 g). However, data collected during the single-laboratory validation (SLV) demonstrate the method is suitable for the analysis of samples containing up to 3 g/100 g GOS (6). The repeatability and reproducibility for six of the samples meet the requirements of SMPR 2014.003 with values of RSD_r_ between 1.4 and 4.7% and RSD_R_ between 8.1 and 11.6%. The precision achieved for one sample, adult nutritional RTF (PBK-00007), does not meet the SMPR requirement with an RSD_r_ of 9.9% and RSD_R_ of 12.1%. This sample is an RTF product and has deposits at the bottom of the bottle. It is necessary to keep the sample under constant agitation during sampling. It is possible that poor homogenization of the product when taking the test portion is responsible for the poor repeatability results. One laboratory (Laboratory 10) was frequently identified as an outlier (five out of seven results). After investigation, the study director realized that the scale of the fluorescence detector was not well adapted for the analysis. It is recommended to adapt the FLD settings to ensure the signal from the highest GOS peak is between 50 and 80% of the maximum scale on the FLD. Laboratory 10 adapted the FLD settings such that the lactose peak was between 50 and 80% of the maximum scale, hence the GOS signals were all much smaller than for the other participants. Apart from the five outliers from Laboratory 10, only two additional outliers were removed prior to statistical analysis based on Cochran and Grubbs tests. Laboratory 5 was an outlier according to a Cochran test on infant formula powder (PBK-0006) and Laboratory 11 was an outlier according to a single Grubbs test on infant formula powder (PBK-0003). Completely eliminating Laboratory 10 from the data set and re-analyzing the data did not have a major impact on the results, and no additional outliers were identified (data not reported). Horwitz ratio (HorRat) values were also estimated and ranged from 1.9 to 3.0, which is higher than generally acceptable. However, even if applicable to most chemical methods, HorRat is not necessarily applicable to methods with polymeric analytes such as galactooligosaccharides (11), therefore interpretation of the data should be made with caution.

**Table 6.** Collaborative study results

Target (SMPR 2014.003):			≤6%	≤12%	Horwitz ratio^e^	Meets SMPR
Sample description	*n^a^*	Overall mean, g/100g*^b^*	RSD_r_, %	RSD_R_, %		
IF powder with GOS/HMO (PBK-00001)^c^	12 (1)	0.594	2.6	10.3	2.4	Yes
IF powder (PBK-00002)^c^	13 (0)	0.688	2.2	9.7	2.3	Yes
IF powder with GOS/FOS (PBK-00003)^c^	11 (2)	0.616	2.3	8.1	1.9	Yes
RTF formula with GOS/FOS (PBK-00004)^d^	12 (1)	0.858	2.1	9.5	2.3	Yes
Organic RTF formula with probiotics (PBK-00005)^d^	12 (1)	0.316	4.7	10.9	2.3	Yes
IF powder with partially hydrolyzed protein (PBK-00006)^c^	11 (2)	0.236	1.4	11.6	2.3	Yes
RTF adult nutritional (PBK-00007)^d^	11 (2)	0.838	9.9	12.1	3.0	No

a *n* = Number of laboratories considered in the evaluation (number of laboratories with data removed as outliers).

b Results are reported in g/100 g of product as consumed (reconstituted or RTF).

c Reconstitution rate: 25 g product + 200 g water.

d RTF = Ready-to-feed.

e Horwitz ratio is not necessarily applicable to methods with polymeric analytes such as galactooligosaccharides (11).

**Table 7.** Results per sample and laboratory (powder samples)^a^

	PBK-00001 infant formula (1)		PBK-00002 infant formula (2)		PBK-00003 infant formula (3)		PBK-00006 infant formula (4)	
Lab code	Sample 21–002	Sample 21–006	Sample 21–004	Sample 21–012	Sample 21–010	Sample 21–013	Sample 21–008	Sample 21–014
1	0.623	0.631	0.719	0.695	0.613	0.636	0.244	0.249
2	0.609	0.585	0.675	0.642	0.640	0.613	0.228	0.231
3	0.592	0.587	0.649	0.663	0.627	0.623	0.238	0.239
4	0.627	0.628	0.697	0.694	0.632	0.637	0.265	0.263
5	0.631	0.651	0.739	0.709	0.666	0.644	0.310^b^	0.333^b^
6	0.517	0.512	0.610	0.625	0.552	0.565	0.212	0.217
7	0.600	0.613	0.702	0.748	0.654	0.663	0.271	0.277
8	0.563	0.599	0.657	0.658	0.603	0.584	0.227	0.225
9	0.743	0.714	0.833	0.806	0.681	0.724	0.274	0.278
10	0.932^c^	0.943^c^	0.792	0.796	0.657^b^	0.337^b^	0.315^b^	0.697^b^
11	0.561	0.518	0.649	0.649	0.572^b^	0.730^b^	0.220	0.221
12	0.524	0.501	0.601	0.599	0.543	0.545	0.193	0.186
13	0.562	0.566	0.626	0.643	0.554	0.562	0.218	0.211
14	Unable to report results							

a Total GOS results in g/100 g product as consumed (reconstituted).

b Outlier according to Cochran test.

c Outlier according to single Grubbs test.

**Table 8.** Results per sample and laboratory (RTF samples)^a^

	PBK-00004 infant formula—RTF		PBK-00005 infant formula—RTF		PBK-00007 adult nutritional—RTF	
Lab code	Sample 21–001	Sample 21–009	Sample 21–005	Sample 21–011	Sample 21–003	Sample 21–007
1	0.810	0.839	0.344	0.308	0.931	0.865
2	0.985	1.00	0.349	0.335	1.36^c^	1.23^c^
3	0.950	0.926	0.355	0.304	0.760	0.703
4	0.882	0.879	0.362	0.360	0.936	0.942
5	0.778	0.826	0.346	0.324	0.850	0.622
6	0.787	0.780	0.277	0.267	0.797	0.815
7	0.849	0.893	0.317	0.336	0.888	0.907
8	0.841	0.850	0.314	0.302	0.814	0.823
9	0.981	1.02	0.357	0.364	1.43^b^	0.814^b^
10	1.19^b^	0.835^b^	0.534^c^	0.576^c^	1.12	0.853
11	0.773	0.775	0.273	0.282	0.698	0.835
12	0.776	0.787	0.284	0.278	0.845	0.805
13	0.812	0.800	0.274	0.273	0.821	0.799
14	Unable to report results					

a Total GOS results in g/100 g product as consumed (reconstituted).

b Outlier according to Cochran test.

c Outlier according to single Grubbs test.

### Determination of Dietary Fiber Contribution

Although GOS are non-digestible, the disaccharide portion of the GOS is excluded from being a component of dietary fiber, which is defined either as carbohydrate polymers with 10 or more monomeric units, or as carbohydrate polymers with three or more monomeric units. In countries adopting the definition with 10 or more monomeric units, none of the GOS will qualify as fiber. However, most countries have adopted the definition with three or more monomeric units, therefore the GOS having a degree of polymerization (DP) ≥3 will qualify. Since this method enables the separation of the GOS disaccharides from the GOS trisaccharides and above, all laboratories were also asked to process the data and report the content of GOS having a DP ≥3 (Tables 9–11). In this case, although the within-lab performance of the method compared well to the performance on total GOS, the interlaboratory reproducibility was much higher (9.8–22%). Although this performance is poor compared to that of the total GOS, it is comparable to the performance of dietary fiber methods which typically have RSD_R_ in the range of 2–20% (14).

**Table 9.** Collaborative study results for GOS with DP ≥3

Sample description	*n^a^*	Overall mean, g/100g*^b^*	RSD_r_, %	RSD_R_, %
IF powder with GOS/HMO (PBK-00001)^c^	13 (0)	0.507	2.4	12.7
IF powder (PBK-00002)^c^	13 (0)	0.565	2.8	12.5
IF powder with GOS/FOS (PBK-00003)^c^	12 (1)	0.401	2.3	11.0
RTF formula with GOS/FOS (PBK-00004)^d^	11 (2)	0.613	1.7	10.8
Organic RTF formula with probiotics (PBK-00005)^d^	13 (0)	0.219	3.5	19.1
IF powder with partially hydrolyzed protein (PBK-00006)^c^	12 (1)	0.161	2.2	21.6
RTF adult nutritional (PBK-00007)^d^	11 (2)	0.550	8.2	9.8

a Number of laboratories considered in the evaluation (number of laboratories with data removed as outliers).

b Results are reported in g/100 g of product as consumed (reconstituted or RTF).

c Reconstitution rate: 25 g product + 200 g water.

d RTF = Ready-to-feed.

**Table 10.** Results of GOS with DP ≥3 per sample and laboratory (powder samples)^a^

	PBK-00001 infant formula		PBK-00002 infant formula		PBK-00003 infant formula		PBK-00006 infant formula	
Lab code	Sample 21–002	Sample 21–006	Sample 21–004	Sample 21–012	Sample 21–010	Sample 21–013	Sample 21–008	Sample 21–014
1	0.535	0.537	0.602	0.581	0.398	0.421	0.159	0.161
2	0.505	0.488	0.562	0.533	0.425	0.404	0.147	0.151
3	0.488	0.506	0.542	0.566	0.418	0.415	0.162	0.160
4	0.515	0.519	0.579	0.579	0.414	0.416	0.167	0.166
5	0.568	0.573	0.647	0.660	0.465	0.445	0.231	0.240
6	0.437	0.431	0.515	0.525	0.358	0.360	0.131	0.133
7	0.530	0.532	0.609	0.652	0.452	0.467	0.198	0.204
8	0.487	0.520	0.562	0.574	0.411	0.397	0.163	0.161
9	0.643	0.623	0.725	0.700	0.490	0.499	0.190	0.194
10	0.568	0.570	0.448	0.484	0.378^b^	0.174^b^	0.179^b^	0.406^b^
11	0.422	0.385	0.490	0.492	0.329	0.477	0.133	0.133
12	0.437	0.419	0.503	0.495	0.353	0.343	0.113	0.105
13	0.469	0.472	0.522	0.543	0.362	0.364	0.137	0.130
14	Unable to report results							

a GOS results in g/100 g product as consumed (reconstituted).

b Outlier according to Cochran test.

c Outlier according to single Grubbs test.

**Table 11.** Results of GOS with DP ≥3 per sample and laboratory (RTF samples)^a^

	PBK-00004 infant Formula—RTF		PBK-00005 infant Formula—RTF		PBK-00007 adult nutritional—RTF	
Lab code	Sample 21–001	Sample 21–009	Sample 21–005	Sample 21–011	Sample 21–003	Sample 21–007
1	0.583	0.589	0.225	0.205	0.626	0.569
2	0.704	0.713	0.223	0.214	0.796^c^	0.715^c^
3	0.694^b^	0.634^b^	0.215	0.219	0.581	0.552
4	0.617	0.615	0.230	0.229	0.600	0.603
5	0.580	0.614	0.253	0.241	0.544	0.463
6	0.546	0.548	0.173	0.167	0.521	0.543
7	0.627	0.652	0.225	0.237	0.605	0.638
8	0.625	0.625	0.221	0.212	0.559	0.555
9	0.741	0.759	0.252	0.253	1.065^b^	0.552^b^
10	0.739^b^	0.505^b^	0.311^b^	0.335^b^	0.563	0.403
11	0.554	0.555	0.183	0.188	0.477	0.549
12	0.541	0.553	0.174	0.171	0.554	0.521
13	0.581	0.569	0.174	0.173	0.546	0.526
14	Unable to report results					

a GOS results in g/100 g product as consumed (reconstituted).

b Outlier according to Cochran test.

c Outlier according to single Grubbs test.

### Collaborator Comments

For the purpose of running the MLT, all collaborators were provided with an Excel spreadsheet to help them calculate the GU value for each peak in the chromatogram and thus assign the mass. The spreadsheet also included GU ranges for lactose and for LNnT before and after β-galactosidase treatment. Laboratoriess 1 and 3 had to make some adaptation to the GU values assigned to lactose and/or LNnT after β-galactosidase treatment to ensure correct identification of those peaks.

For reconstitution of powdered samples, the method recommends reconstituting samples by adding water and stirring at 70°C for 25 min. Laboratory 4 made additional tests by reconstituting with water at 50°C and stirring for only a few minutes and found no difference in the results, thus they proposed that the reconstitution could be simplified without impacting the results. This approach will speed up sample preparation. It was not introduced in the Final Method since it was not tested during the MLT. Laboratories wishing to follow such an approach should verify equivalence in their laboratory before implementation.

The method employs a dextran ladder to assign GU values to RRTs. In the method, a plot of GU versus RRT is made, and a third-order polynomial is fitted to the data. Laboratory 7 proposed using a plot of log GU versus RRT instead; using such an approach, it is possible to fit a second-order polynomial to the data, or achieve an improved fit of the third-order polynomial. They also noted that it is important only to include GU 1–6 when making the plot if the log transformation is not performed. Plotting the log of GU versus RRT seems like a good recommendation, however, laboratories would have to control if the final peak assignments remain correct before implementation.

Laboratory 7 performed analysis of practice samples using both the dextran ladder and using MS to assign peak masses. They noted the two approaches gave comparable results and, thus, continued with the MLT using only the dextran ladder.

Laboratory 9 attempted to run the practice samples using the Agilent AdvanceBio Glycan Mapping column (150 mm × 4.6 mm, 2.7 μm); however, to achieve similar separation the gradient conditions would need to be altered, and they therefore continued with the Waters BEH Glycan column.

Laboratory 13 initially ran the practice samples using both MS and the dextran ladder to assign peak masses. Using MS, they observed a trisaccharide eluting after only 5.1 min which is much earlier than the expected trisaccharide retention times (15–30 min), and earlier than the expected retention time of GOS disaccharides. After some discussions and swapping of chromatograms with the study director, it was concluded that this signal did not originate from the GOS (or from the samples). Laboratory 13 continued with the analysis applying the dextran ladder to assign peak masses and ignoring the presence of this unusual signal, the source of which remains a mystery (nothing similar has been observed in the study director’s laboratory, and no other laboratories reported anything similar).

### Study Director Comments

The analysis of GOS following the procedure outlined in AOAC Method **2021.01** is not trivial. Several labs required guidance during the setup of the method. When setting up this method for the first time, we would recommend starting using different GOS ingredients (preferably with a mass spectrometer in parallel with the FLD) so the analyst gets experience with different GOS chromatograms and processing the data before moving on to complex matrixes. Such an approach can also be used to adapt the calibration of a column with the dextran ladder in case an alternative column with different selectivity is employed.

Key points to control when running the method are: *(1)* Check the purity and moisture content of the maltotriose and correct for those when constructing the calibration curve. *(2)* Check the integration of the signals in the chromatogram as the chromatograms are not easily integrated using the automated tools built into chromatographic software. *(3)* Ensure that only peaks with a S/N of ≥10 are included for data processing (including smaller peaks can introduce significant errors).

The proposals of Laboratory 4 to simplify sample reconstitution and of Laboratory 7 to plot log GU versus RRT are worthy of consideration when setting up the method as it could simplify the analytical and data processing processes respectively.

## Conclusions

The method described in AOAC Method **2021.01** achieved good results generally in line with the requirements of AOAC SMPR 2014.003 during both the SLV (6) and MLT. Since the performance on the adult nutritional sample did not meet the SMPR 2014.003, the method has not been adopted for application to adult nutritional products (further investigations on such matrixes are required).

## Supplementary Material

qsae031_Supplementary_Data
